# Supplementary material for: Oxidation of SQSTM1/p62 mediates the link between redox state and protein homeostasis
Source: Nat Commun. 2018 Jan 17;9:256. doi: 10.1038/s41467-017-02746-z (PMC5772351; doi:10.1038/s41467-017-02746-z)
Supplement: Supplementary file 1 — Supplementary Information [file 41467_2017_2746_MOESM1_ESM.pdf]

Supplementary Information

Supplementary Figures

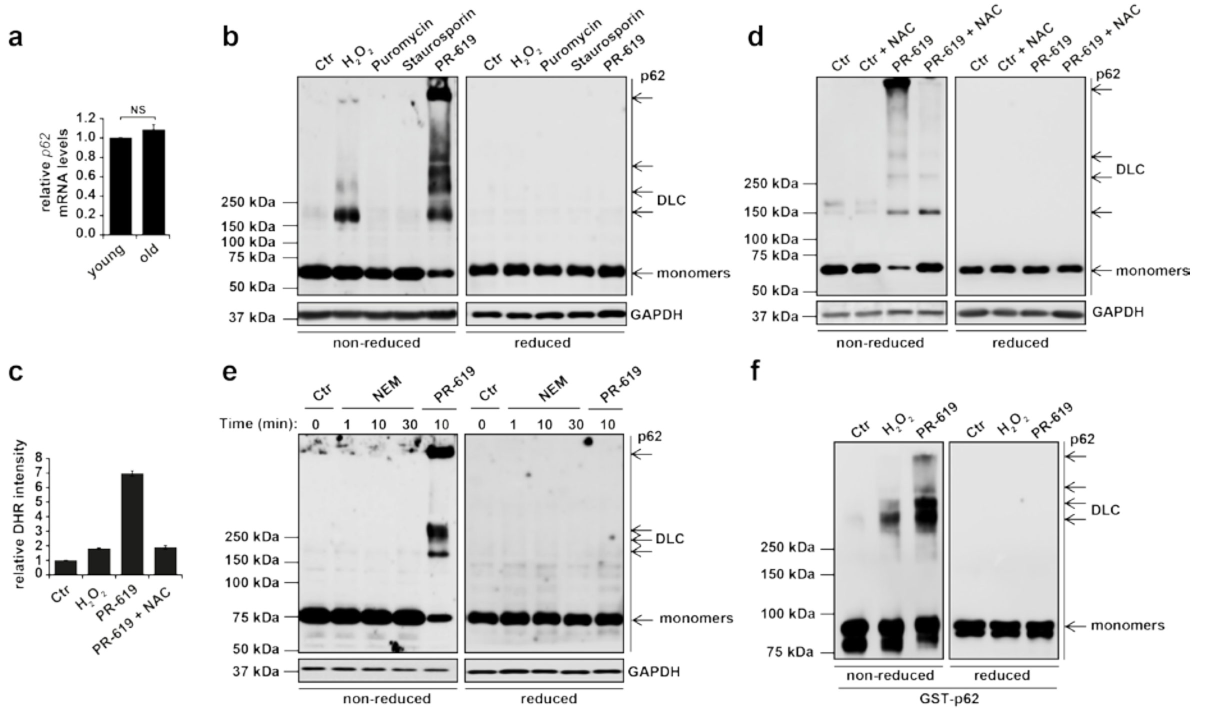

Supplementary Fig. 1. Oxidative stress conditions induce the formation of p62 DLC. (**a**) p62 mRNA levels in brain tissue, from young (3 months) and old (24 months) mice were analysed by qPCR, Pkg1 was used as a loading control (n=5 per group; NS = not significant (unpaired t-test). (**b**) HeLa cells were treated with H_2O_2 (5mM, 1 min), puromycin (10µg/ml, 3 hours), staurosporin (0.5µM and Z-VAD 6.6µM, 3 hours) and PR-619 (10µM, 10 min), lysed and subjected to immunoblot analysis for p62 in reducing (2.5% β-ME) and non-reducing conditions. DLC indicate disulphide-linked conjugates. Arrows indicate the positions of monomeric proteins and DLC. **(c)** HeLa cells were treated with PR-619 (10µM, 10 minutes) in the presence or absence of the antioxidant N-acetylcysteine (NAC, 5mM). Oxidation levels were assessed by staining cells with dihydrorhodamine 123 (DHR) and analysed by fluorescence-activated cell sorting (FACS). Data were normalised, error bars represent standard deviation. (**d**) Cells were treated as in (**c**) then lysed and subjected to immunoblot analysis for endogenous p62 in reducing and non-reducing conditions. (**e**) Inhibition of deubiquitylases does not induce p62 DLC. HeLa cells were treated with N-ethylmaleimide

(NEM, 50mM) at different time points and PR-619 (50μM), then lysed and subjected to immunoblot analyses for endogenous p62 in reducing and non-reducing conditions. (**f**) p62 forms DLC in response to oxidation *in vitro*. GST-p62 was purified from *E. coli*, treated with H_2O_2 (3mM, 5min) or PR-619 (50μM, 5min) and immunoblotted for p62 in reducing and non-reducing conditions. All blots and graphs (except (a)) represent an average of at least two independent experiments and error bars represent standard deviations.

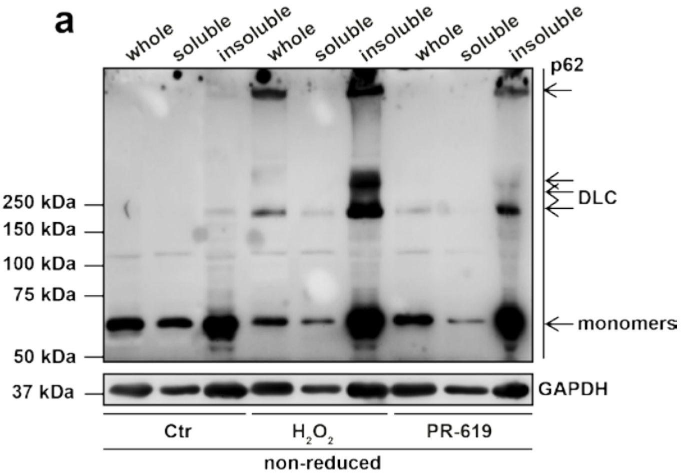

Supplementary Fig. 2. DLC of p62 are found in insoluble fractions. (**a**) Oxidative stress induces a shift of p62 to the insoluble fraction. HeLa cells were treated with H_2O_2 (5 mM, 1 min) and PR-619 (10 μM, 10 min), lysed and subjected to ultracentrifugation to separate the soluble and insoluble fractions. Whole cell, soluble and insoluble fractions were then immunoblotted for endogenous p62. In control cells, p62 is distributed between supernatant and pellet fractions in agreement with previous findings that the cellular pool of p62 exists between soluble and aggregated states [1]. Treatment with H_2O_2 or PR-619 induced a shift to the insoluble state, with the majority of DLC found in the pellet. Arrows indicate the positions of monomeric proteins and DLC. Blot is a representative example of three independent experiments.

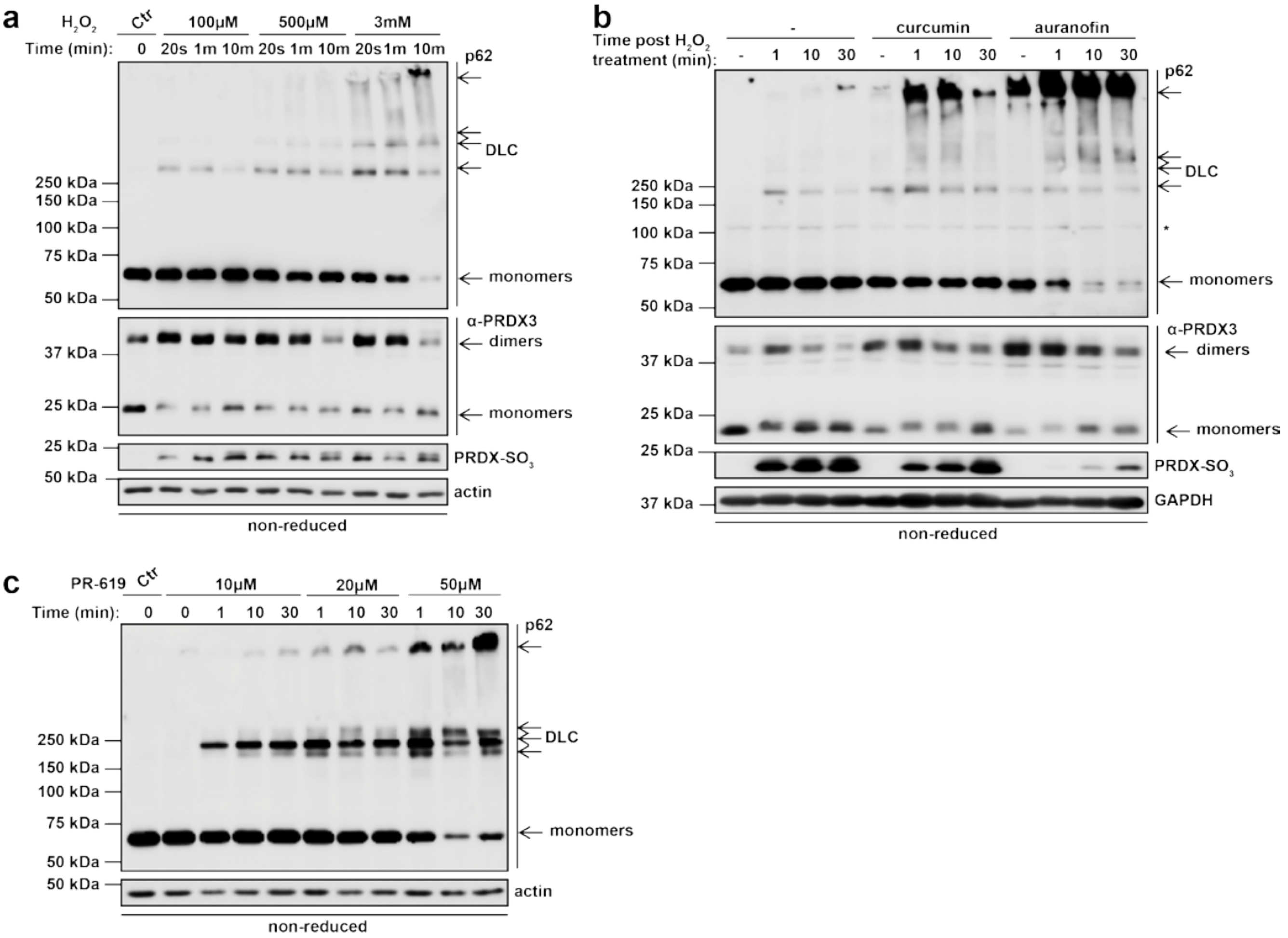

Supplementary Fig. 3. H_2O_2 and PR-619 show different kinetics of DLC formation and clearance. (**a**) H_2O_2 triggers rapid but reversible p62 DLC formation in a concentration-dependent manner. HeLa cells were treated with increasing concentrations of H_2O_2 for different time points as shown, then lysed and immunoblotted for p62, PRDX3 and PRDX-SO_3. (**b**) Thioredoxin reductase is required to resolve p62 DLC. HeLa cells were pre-treated with thioredoxin reductase inhibitors, curcumin (50μM) or auranofin (5μM) for 30 min, then treated with H_2O_2 (500μM) at different time points as shown. Cell were then lysed and immunoblotted for p62, PRDX3 and PRDX-SO_3. The reduced disulphide-linked PRDX dimers and increased PRDX-SO_3 in response to H_2O_2 indicates the curcumin and auranofin are inhibiting Trx. (**c**) PR-619 induces sustained p62 DLC. HeLa cells were treated with increasing concentrations of PR-619 at different time points as shown, then lysed and subjected to immunoblot analysis for endogenous p62. Arrows indicate the positions of

monomeric proteins and DLC. All blots are representative of at least two independent experiments.

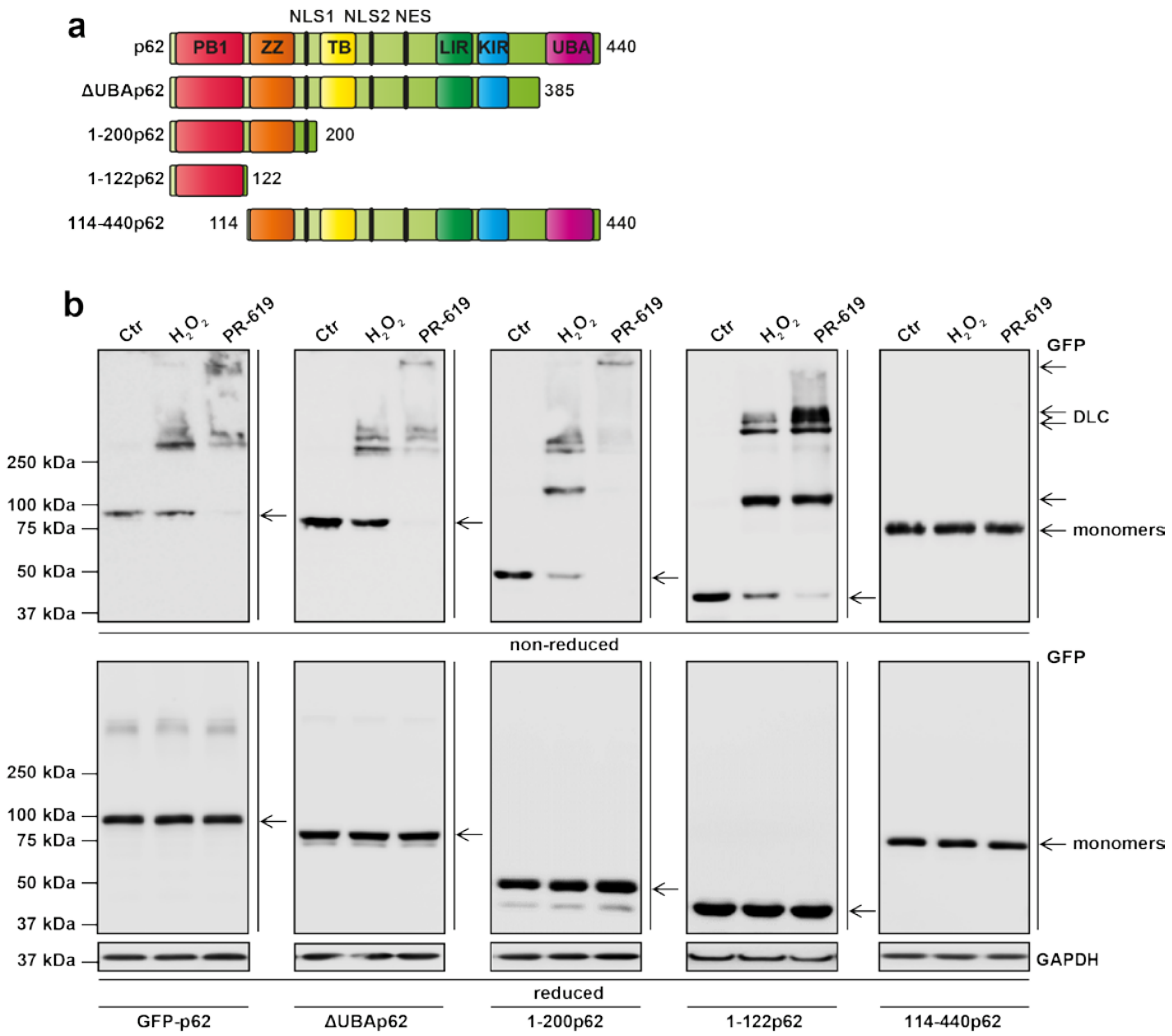

Supplementary Fig. 4. N-terminus of p62 is required for DLC formation. (**a**) Diagram of p62 deletion constructs used in this study. (**b**) HeLa cells were transfected with different GFP-tagged p62 constructs, treated with H_2O_2 (1mM, 1 min) and PR-619 (10μM, 10 min) and subjected to immunoblot analysis for GFP-tagged p62 in reducing (2.5% β-ME) and non-reducing conditions. PB1: Phox and Bem1P domain, ZZ: zinc finger domain, NLS: nuclear localisation signal, TB: TRAF-binding domain, NES: nuclear export signal, LIR: LC3-interacting region, KIR: KEAP1-interacting region, UBA: ubiquitin-binding domain. All blots are representative of at least two independent experiments.

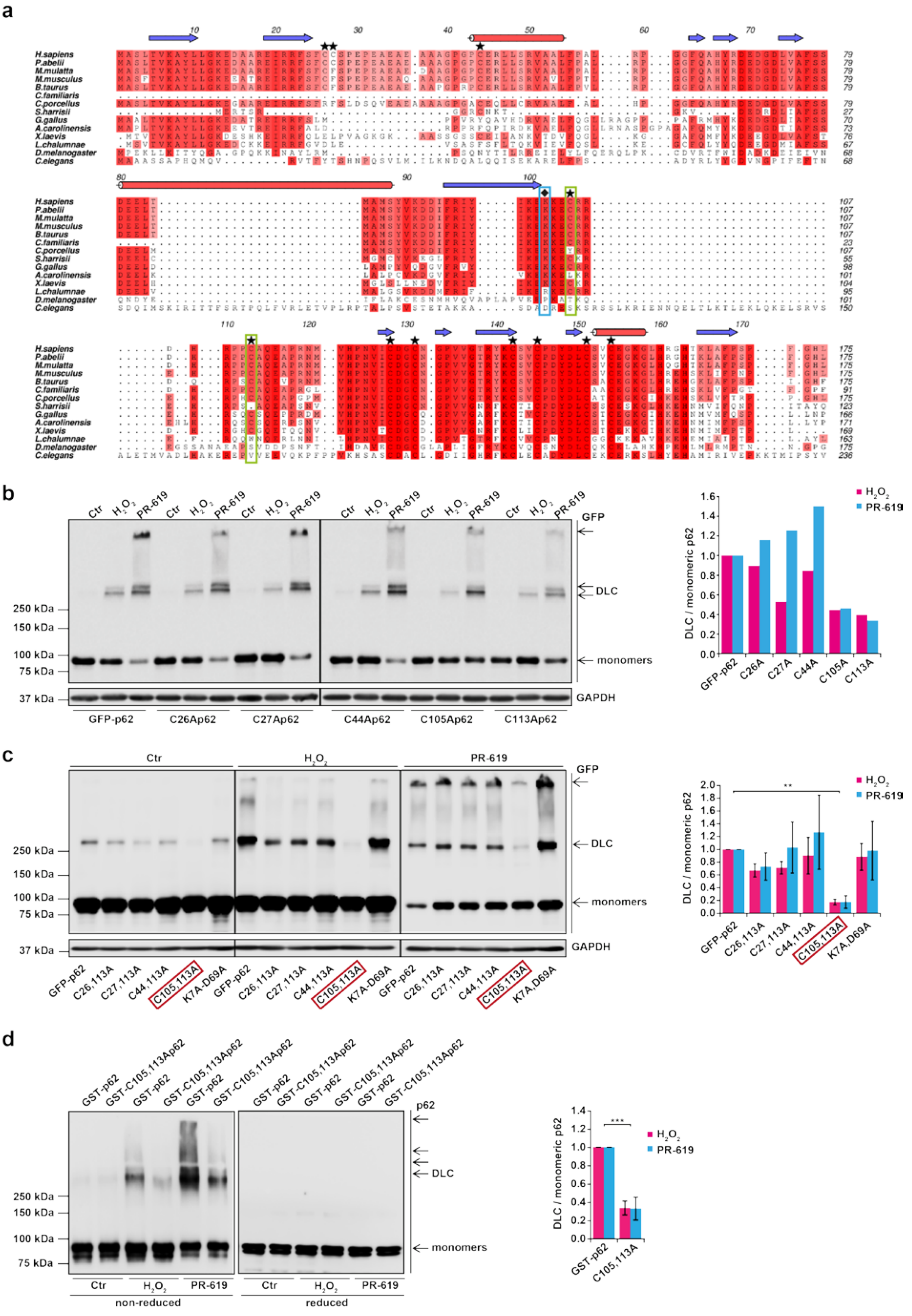
a
H.sapiens
P.abelii
M.mulatta
M.musculus
B.taurus
C.familiaris
C.porcellus
S.harrisii
G.gallus
A.carolinensis
X.laevis
L.chalumnae
D.melanogaster
C.elegans
b
Ctr
H2O2
PR-619
GFP
DLC
monomers
GAPDH
250 kDa
150 kDa
100 kDa
75 kDa
37 kDa
GFP-p62
C26Ap62
C27Ap62
C44Ap62
C105Ap62
C113Ap62
DLC / monomeric p62
GFP-p62
C26A
C27A
C44A
C105A
C113A
c
Ctr
H2O2
PR-619
GFP
DLC
monomers
GAPDH
GFP-p62
C26,113A
C27,113A
C44,113A
C105,113A
K7A-D69A
K7A,D69A
DLC / monomeric p62
**
d
GST-p62
GST-C105,113Ap62
p62
DLC
monomers
Ctr
H2O2
PR-619
non-reduced
reduced
DLC / monomeric p62
***
GST-p62
C105,113A

Supplementary Fig. 5. Cysteine residues 105 and 113 are required for p62 DLC formation. (**a**) C105 and C113 are the most conserved cysteines in the 1-122 region of p62. Multiple alignments of p62 sequences (amino acids 1-200) in different species. Increasing conservation is shown by light-to-dark red; cysteine residues are indicated by stars, whilst the position of an ALS-related mutation (K102E) by a diamond. Secondary structure is indicated by lines above the sequence alignment with β-sheets and α-helices shown as blue arrows and red cylinders, respectively. (**b**) Mutations of C105 and C113, but no other single cysteine residue impair the formation of p62 DLC. HeLa cells were transfected with GFP-tagged wild-type or different p62 cysteine to alanine mutants as shown, treated with H_2O_2 (1mM, 1 min) and PR-619 (10μM, 10 min) and subjected to immunoblot analysis for GFP-tagged p62. The ratio of DLC to monomeric p62 was quantified and normalised to the untreated. (**c**) C105 and C113 of p62 are important for the formation of DLC. HeLa cells were transfected with GFP-tagged wild type or different double cysteine mutants of p62, treated as in (B) and subjected to immunoblot analysis for GFP-tagged p62 and quantified. The p62 double mutant C105A,C113A is highlighted by a red box. (**d**) GST-p62 and GST-C105,113Ap62 was purified from *E. coli*, treated with H_2O_2 (3mM, 5min) and PR-619 (50μM, 5min) and immunoblotted for p62 in reducing and non-reducing conditions. Error bars represent s.e.m., ***P*<0.01, *** *P*<0.005 (unpaired t-test) from three independent repeats. Arrows indicate the positions of monomeric and oligomeric p62. Blots and quantification in B are representative of at least two independent experiments.

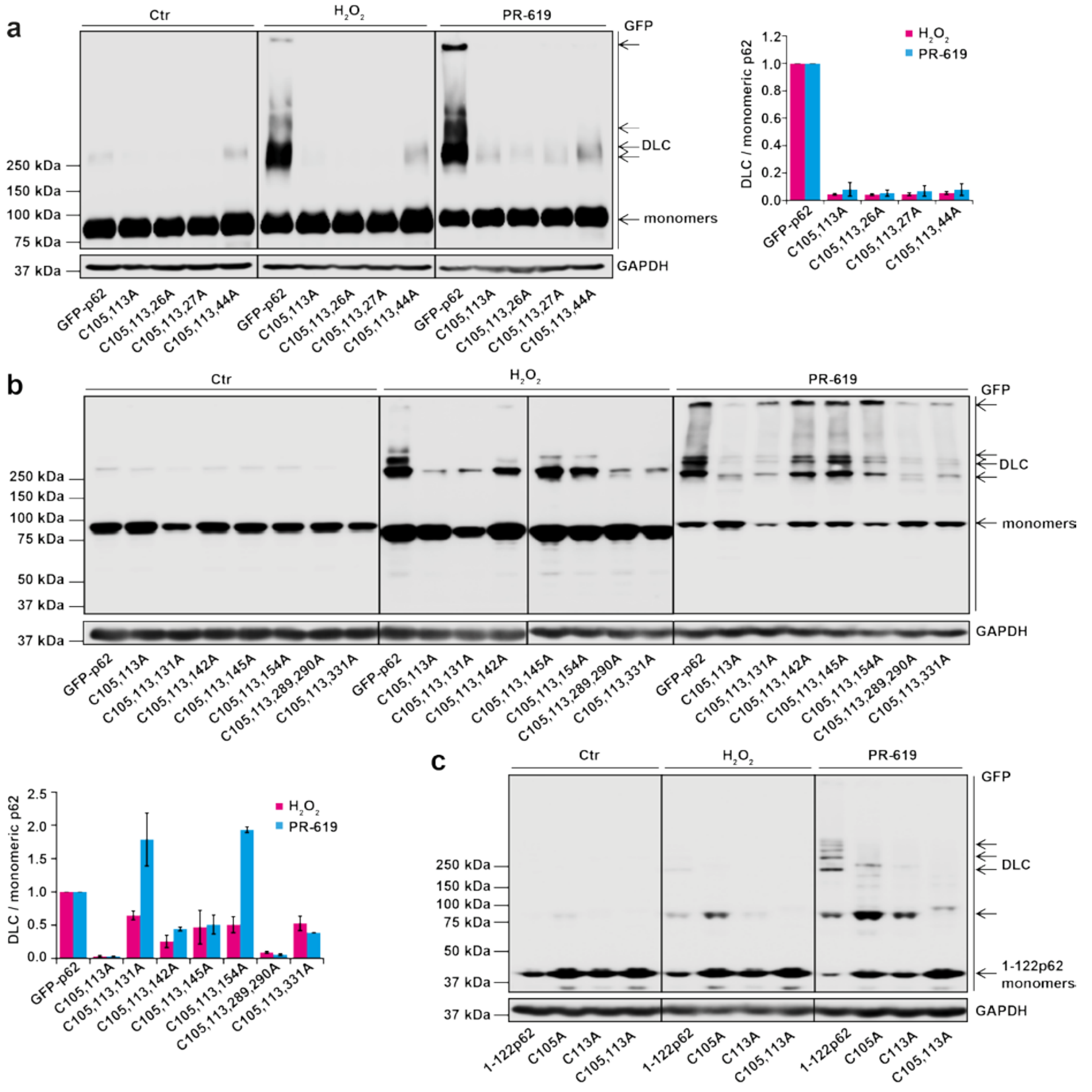

Supplementary Fig. 6. There is no additive effect on suppression of DLC formation with additional cysteine residue mutations. As low-level residual oxidation of the C105A,C113A mutant remained, we attempted to identify additional Cys residues which could be involved in this process by performing the third round of mutagenesis using C105A,C113A as a template. In particular, we substituted Cys residues in the N-terminal region (**a**) and several others which have the highest degree of conservation amongst the species we investigated (**b**)**.** GFP-tagged wild-type or different, triple Cys mutants (produced using the C105, C113A backbone) of p62 either in the N-terminal region (**a**) or along its sequence (**b**) were expressed in HeLa cells as shown. Cells were treated with H_2O_2 (1mM, 1

min) and PR-619 (10μM, 10 min) and subjected to immunoblot analysis for GFP-tagged p62. The ratio of DLC to monomeric p62 was quantified. None of these additional mutations caused any further impairment of DLC formation. Moreover, several new point-mutations (C131A, C142A, C145A and C154A) (b) located in the ZZ-finger domain partially restored the ability of C105A-C113A to form DLC, possibly by exposing additional Cys residues which are normally involved in the chelation of Zn^{2+} ion. (**c**) An important role of C105/C113 (but not other Cys residues within the 1-122 fragment of p62) for the formation of DLC was confirmed using the N-terminal fragment which becomes insensitive to oxidation upon mutagenesis of these residues. HeLa cells were transfected with GFP-tagged N-terminus wild-type or different mutants of p62 (amino acids 1-122), treated as in (a) and subjected to immunoblot analysis for GFP-tagged p62. Arrows indicate the positions of monomeric proteins and DLC. Error bars represent standard deviations. All blots represent an average of at least two independent experiments.

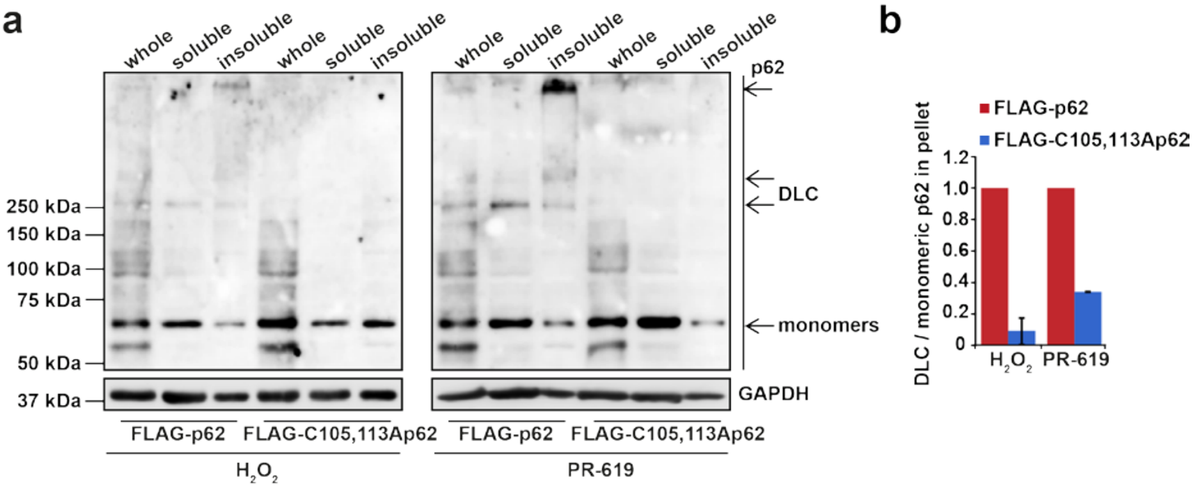

Supplementary Fig. 7. C105A,C113A mutation impairs p62 shift to the insoluble fraction. (**a**) *p62*$^{-/-}$ MEFs stably expressing FLAG-tagged wild-type or C105A,C113A p62 were treated with H_2O_2 (5mM, 1 min) and PR-619 (10μM, 10 min), lysed and subjected to ultracentrifugation to separate the soluble and insoluble fractions. Whole cell, soluble and insoluble fractions were then immunoblotted for endogenous p62. (**b**) The ratio of DLC to monomeric p62 was quantified and normalised to the untreated. Arrows indicate the

positions of monomeric proteins and DLC. Error bars represent standard deviations. All blots represent an average of at least two independent experiments.

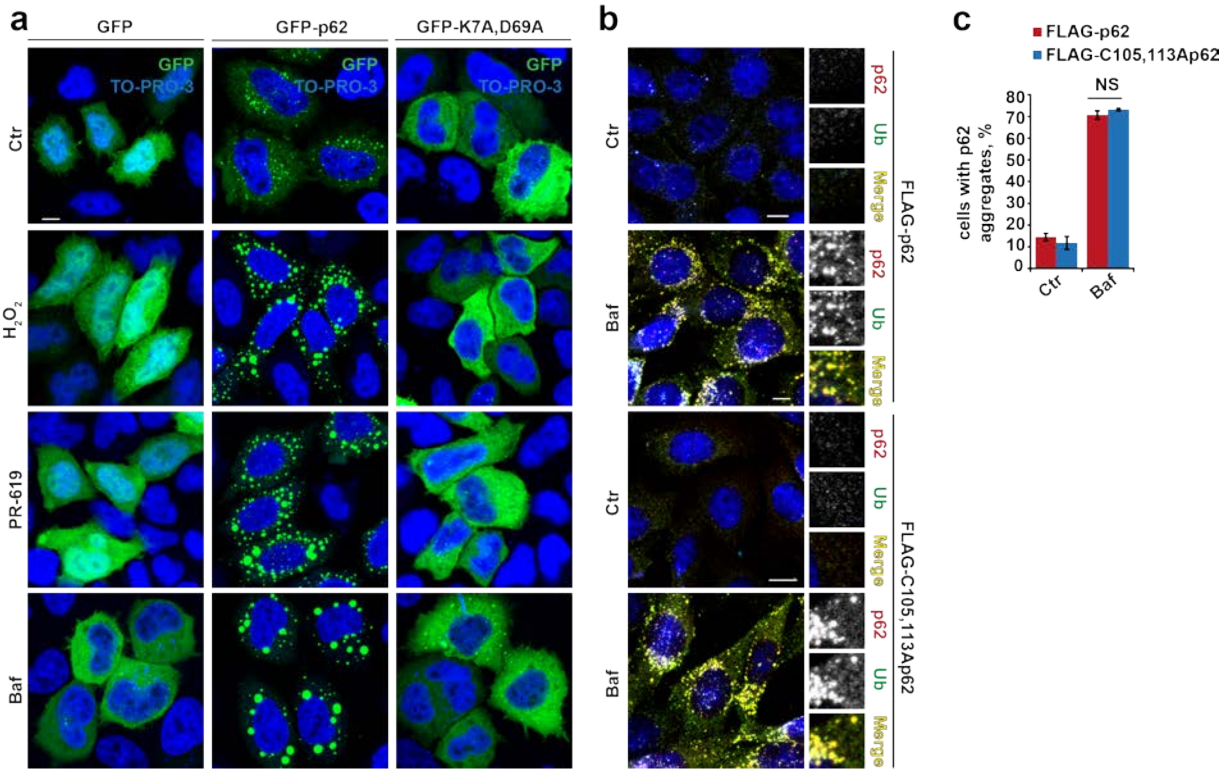

Supplementary Fig. 8. DLC and PB1 domain-mediated interactions cooperate in the formation of ubiquitylated aggregates. (**a**) Loss of the PB1 domain function of p62 prevents the formation of aggregates in both autophagy deficiency and oxidative stress conditions. HeLa cells were transfected with GFP, GFP-tagged wild type or K7A,D69A p62, treated with H_2O_2 (5mM, 1 min) or PR-619 (10µM, 10 min) or with bafilomycin A1 (Baf, 400 nM, 4 hours). Cells were fixed and analysed by confocal microscopy. (**b, c**) Cysteine residues C105 and C113 are not required for aggregation of p62 in conditions of autophagy inhibition. Stably expressing FLAG-tagged wild type and C105A,C113A p62 cell lines were treated with bafilomycin A1 (Baf) as in (a) or left untreated (Ctr), immunostained for ubiquitin and p62 and analysed by confocal microscopy. (**c**) Cells with and without aggregates were counted and their proportion quantified. Nuclei are visualized in merge images with TO-PRO-3 iodide. Graph represents an average of at least three independent experiments and error bars represent s.e.m., NS: not significant (unpaired t-test). Scale bar 10µm.

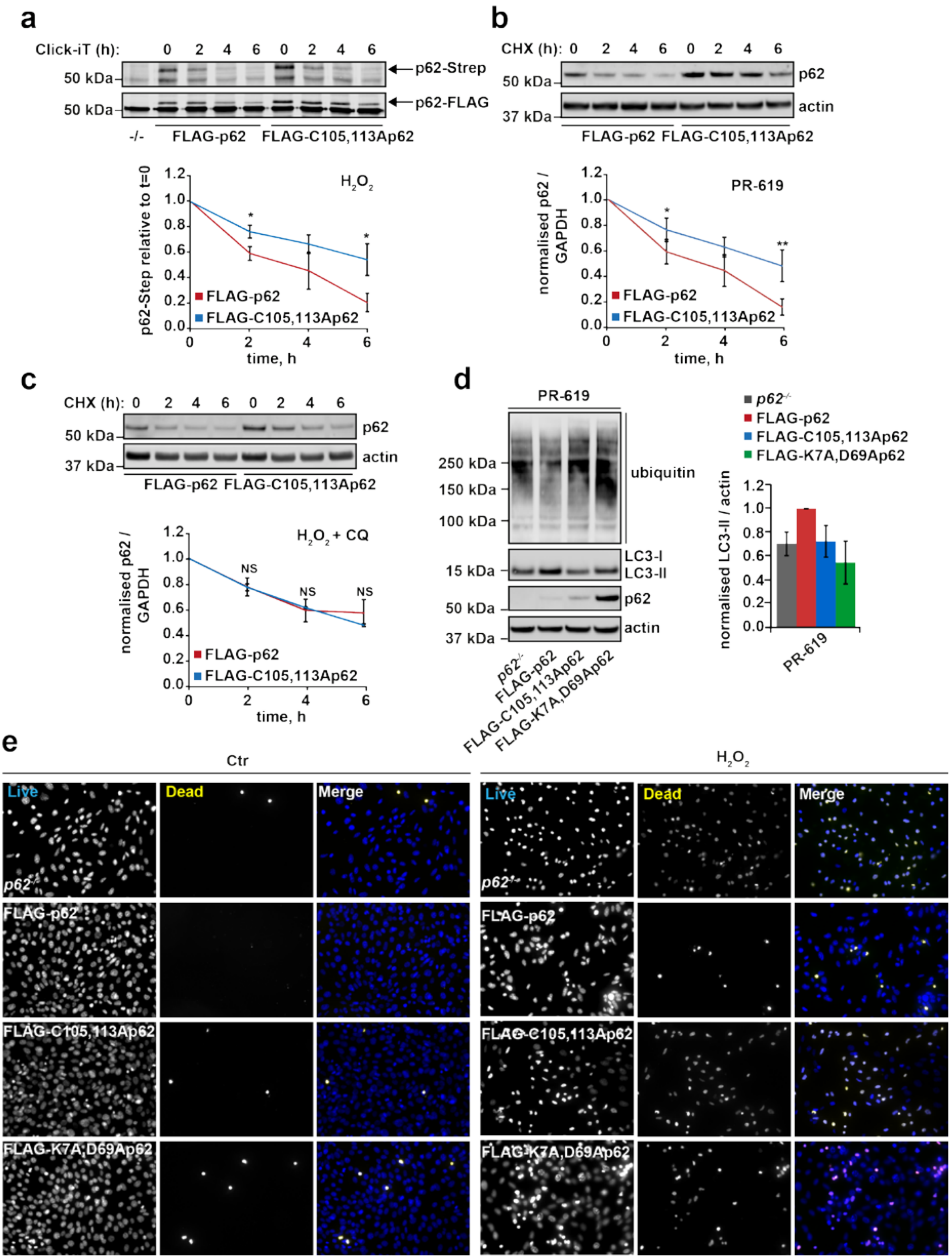

Supplementary Fig. 9. C105A,C113A mutation shows slower autophagic turnover. (**a**) Protein degradation rates of FLAG-p62 and FLAG-C105A,C113Ap62 MEFs were analysed by Click-iT assay where AHA reagent is incorporated into newly synthesised proteins and monitored via biotin pull-down over a chase period of 6 hours. (**b, c**) *p62*$^{-/-}$ MEFs stably

expressing FLAG-tagged wild type or C105A,C113A p62 were treated with cycloheximide (CHX, 50µg/ml) and PR-619 (5µM) **(b)** or CHX, H_2O_2 (1mM) and chloroquine (20µM) **(c)**, lysed at the indicated time post treatment, immunoblotted for p62 and quantified. (**d**) p62 stable cell lines were treated with PR-619 (5µM) for 3 hours, lysed and immunoblotted for LC3, p62 and ubiquitin. (**e**) p62 stable cell lines were maintained in serum free media for five hours either in the presence or absence of H_2O_2 (1mM). % cell death was analysed by ReadyProbes fluorescent dyes. Graph represents an average of at least three independent experiments and error bars represent s.e.m., **P*<0.05, ***P*<0.01, ****P*<0.005. NS = not significant (unpaired t-test).

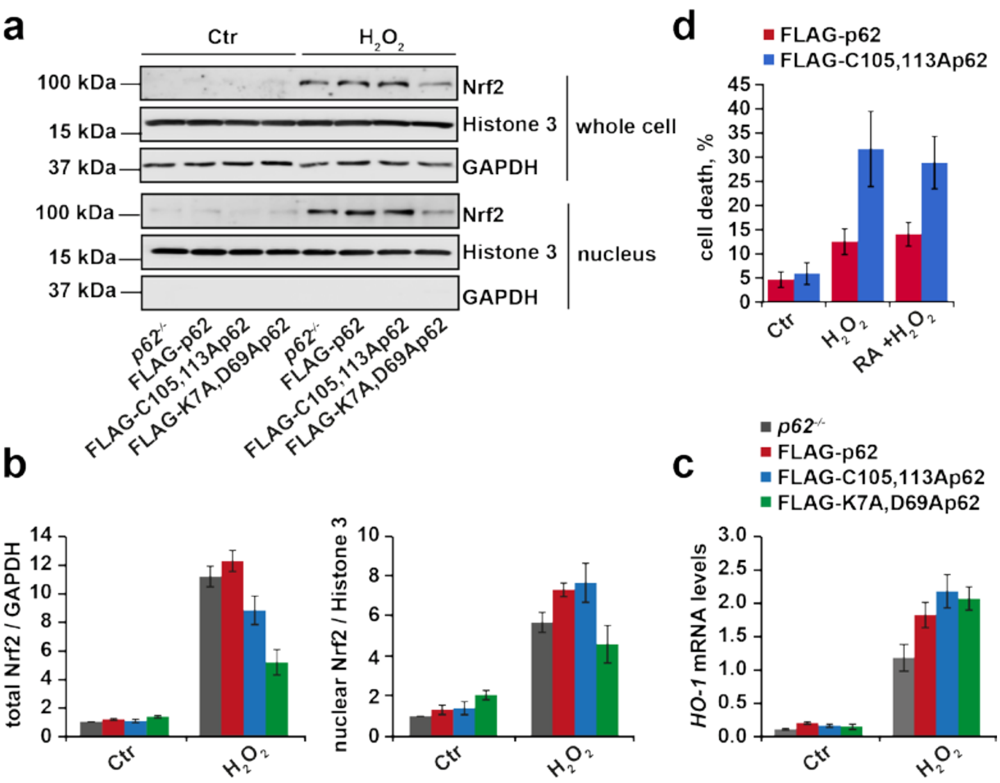

Supplementary Fig. 10. Impaired survival of C105A,C113A p62 expressing cells in oxidative stress conditions is not mediated by an alteration in Nrf2 signalling. (a) *p62*$^{-/-}$ MEFs stably expressing FLAG-tagged wild type, C105A,C113A or K7A,D69A PB1 domain mutant p62 were treated with H_2O_2 (1mM) in serum free media for 5 hours and subjected to a nuclear fractionation followed by immunoblot analysis for Nrf2, Histone 3 and GAPDH and quantified (**b**). **(c)** Cells were treated as in (a) and Nrf2 target gene *HO-1* mRNA levels were

analysed by qPCR, actin was used as a loading control. (d) *p62*$^{-/-}$ MEFs stably expressing FLAG-tagged wild type or C105A,C113A mutant p62 were pre-treated with retinoic acid (RA, 30µM) for one hour in serum free media followed by the same treatment as in (a) with or without retinoic acid (30µM) and % cell death was analysed by ReadyProbes fluorescent dyes (Life Technologies).

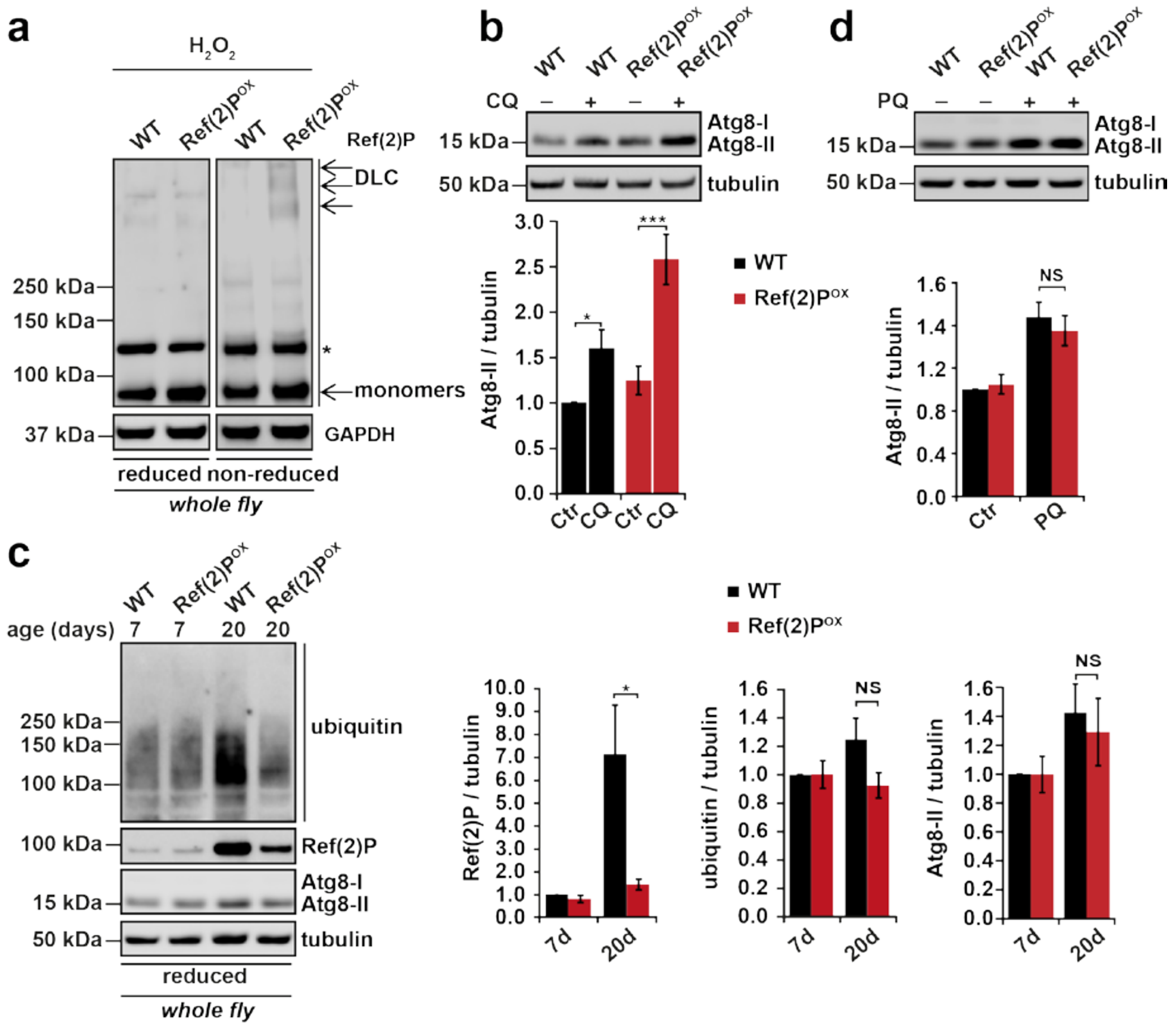

Supplementary Fig. 11. 'Humanised' Ref(2)P^{ox} flies are characterised by increased autophagic flux and reduced age-related protein accumulation. (**a**) Whole fly lysates from young flies treated with H_2O_2 *in vitro* (1mM, five minutes) were analysed by western blot in reducing and non-reducing conditions. (**b**) Flies were fed with chloroquine (2.5mg/ml) for 2 days. Whole fly lysates were immunoblotted for Atg8 and quantified. (**c**) Whole fly lysates from young (7 days) and aged (20 day) flies were immunoblotted for Ref(2)P, ubiquitin and

Atg8, and quantified. (**d**) Wild-type (WT) and Ref(2)P^{ox} flies were treated with paraquat (PQ, 20mM) for 12 h and whole fly lysates were analysed by immunoblot for Atg8 and tubulin and quantified. All graphs represent an average of at least three independent experiments (at least 10 flies per replicate) and error bars represent s.e.m., **P*<0.05, ***P*<0.01, NS: not significant (un-paired t-test).

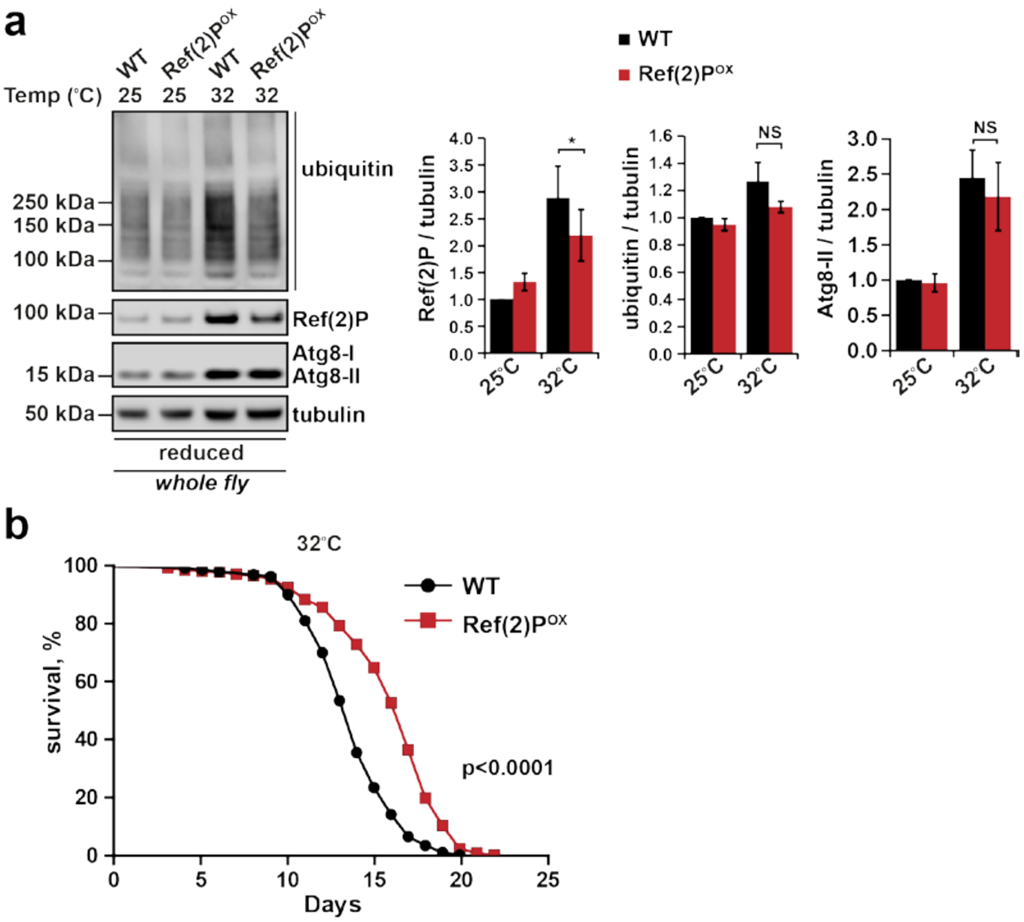

Supplementary Fig. 12. 'Humanised' Ref(2)P^{ox} flies have increased resistance to thermal stress. (**a**) Two day old flies were maintained at 25°C or transferred to 32°C for 2 days. Whole fly lysates were immunoblotted for Ref(2)P, ubiquitin and Atg8, and quantified. (**b**) Combined survival data (of three repeats, 60 flies per group per replicate) of wild-type versus Ref(2)P^{ox} flies at 32°C. Log rank statistics were applied. Blots and quantification represent an average of at least three independent experiments (at least 10 flies per group per replicate) and error bars represent s.e.m., **P*<0.05, NS: not significant (unpaired t-test).

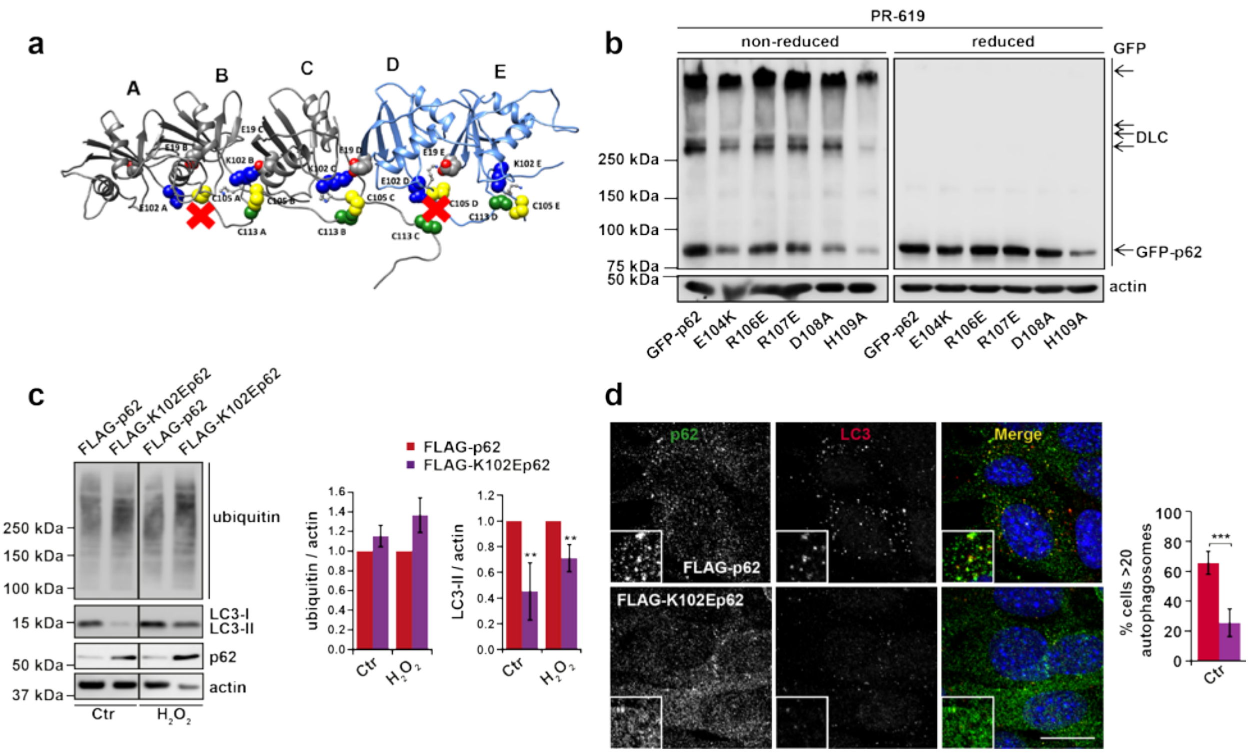

Supplementary Fig. 13. ALS-related mutation of p62 impairs the formation of DLC. (**a**) A hypothetical *in silico* model showing the orientation of the N-terminal PB1 domains (residues 3-125) and the impact of K102E mutation on oligomerisation. Two of the monomers (A and D) are K102E mutants, while monomers B, C and E are wild-type. K102E mutation triggers large conformational changes locally, which affect the position and orientation of C105 residue (yellow) and impair the disulphide bond between C105 and C115 (green) from the previous monomer (e.g. K102E mutation in chain D disrupts the disulphide bond between C105 from the monomer D and C115 from the monomer C. Thus, equimolar mixture of WT and K102E mutation results in the covalently-linked trimers (coloured grey in the figure) and disrupts the larger oligomers. Residue K102 is coloured blue. In wild-type protein it interacts with E19 (highlighted as spheres and coloured by heteroatom). In K102E mutant this favourable electrostatic interaction is disrupted, strong electrostatic repulsion triggers the conformational changes, and the adjacent K103 (highlighted in the monomer D and displayed as balls and sticks) moves towards E19, thus compensating for the loss of favourable interaction, while residue 102 moves outwards. (**b**) Other mutations in the region of K102E do not affect p62 DLC formation. HeLa cells were transfected with GFP-tagged

p62 constructs as shown and treated with PR-619 (10μM, 10 min). Samples were immunoblotted for GFP in non-reducing and reducing conditions. (**c**) The ALS-related mutant K102E of p62 impairs autophagy. *p62*$^{-/-}$ MEFs stably expressing wild-type or K102E were treated with H_2O_2 (1mM for 5 hours) and subject to immunoblotting for ubiquitin, LC3 and p62 and quantified. (**d**) *p62*$^{-/-}$ MEFs stably expressing wild-type or K102E in control conditions were fixed and stained for p62 and LC3 and % cells with >20 autophagosomes was quantified. Arrows indicate the positions of monomeric and DLC p62. All graphs represent an average of at least three independent experiments and error bars represent s.e.m., n=3, **P<0.01, ***P<0.005 (unpaired t-test). Scale bar: 20μm.

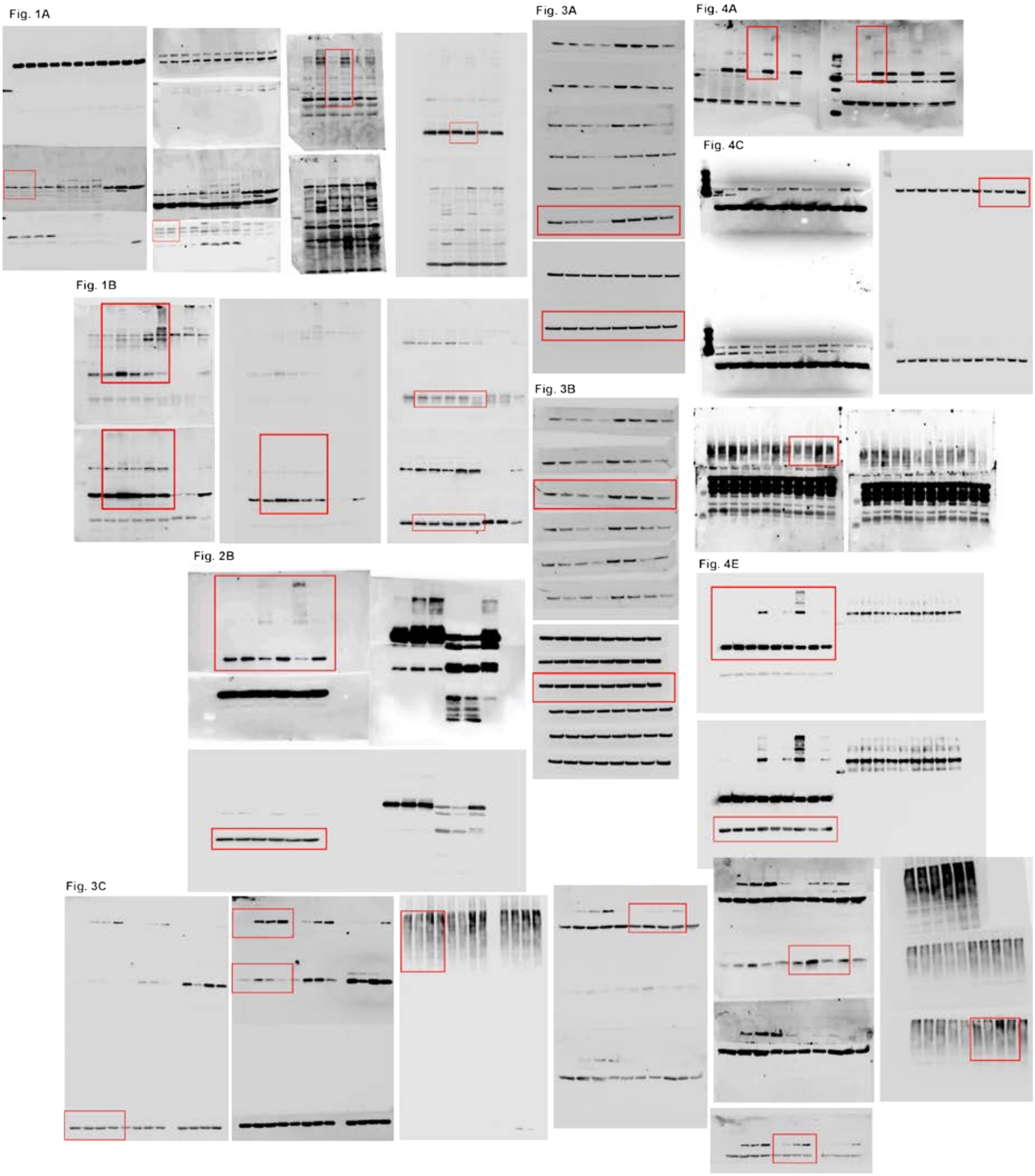

Supplementary Fig. 14. Raw western blot data. Uncropped images of western blots in main figures are shown.

Supplementary Tables

primer	Sequence
K7A forward	5'-CGTCGCTCACCGTGGCGGCCTACCTTCTGG-3'
K7A reverse	5'-CCAGAAGGTAGGCCGCCACGGTGAGCGACG-3'
C26A forward	5'-CCGCTTCAGCTTCGCCTGCAGCCCCGAG-3'
C26A reverse	5'-CTCGGGGCTGCAGGCGAAGCTGAAGCGG-3'
C27A forward	5'-GCTTCAGCTTCTGCGCCAGCCCCGAGCCTG-3'
C27A reverse	5'-CAGGCTCGGGGCTGGCGCAGAAGCTGAAGC-3'
C44A forward	5'-GGTCCGGGACCCGCCGAGCGGCTGCT-3'
C44A reverse	5'-AGCAGCCGCTCGGCGGGTCCCGGACC-3'
D69A forward	5'-CGCACTACCGCGCTGAGGACGGGGA-3'
D69A reverse	5'-TCCCCGTCCTCAGCGCGGTAGTGCG-3'
K102E forward	5’-CCCGCCGGCACTCTTTCTCCTCTTTAATGTAGATTCGGAA-3’
K102E reverse	5’- TTCCGAATCTACATTAAAGAGGAGAAAGAGTGCCGGCGGG-3’
C105A forward	5'-ACATTAAAGAGAAAAAAGAGGCCCGGCGGGACCACCG-3'
C105A reverse	5'-CGGTGGTCCCGCCGGGCCTCTTTTTTCTCTTTAATGT-3'
C113A forward	5'-CCACCGCCCACCGGCTGCTCAGGAGGCG-3'
C113A reverse	5'-CGCCTCCTGAGCAGCCGGTGGGCGGTGG-3'
C128A forward	5'-GCACCCCAATGTGATCGCCGATGGCTGCAATGGG-3'
C128A reverse	5'-CCCATTGCAGCCATCGGCGATCACATTGGGGTGC-3'
C131A forward	5'-GTGATCTGCGATGGCGCCAATGGGCCTGTGGT-3'
C131A reverse	5'-ACCACAGGCCCATTGGCGCCATCGCAGATCAC-3'
C142A forward	5'-GAACCCGCTACAAGGCCAGCGTCTGCCCAG-3'
C142A reverse	5'-CTGGGCAGACGCTGGCCTTGTAGCGGGTTC-3'
C145A forward	5'-CTACAAGTGCAGCGTCGCCCCAGACTACGACTTG-3'
C145A reverse	5'-CAAGTCGTAGTCTGGGGCGACGCTGCACTTGTAG-3'
C151A forward	5'-CCAGACTACGACTTGGCTAGCGTCTGCGAGGG-3'
C151A reverse	5'-CCCTCGCAGACGCTAGCCAAGTCGTAGTCTGG-3'
C154A forward	5'-CAAGCCCTTTCCCTCGGCGACGCTACACAAGTCG-3'
C154A reverse	5'-CGACTTGTGTAGCGTCGCCGAGGGAAAGGGCTTG-3'
C289A/C290A forward	5'-GCTTGCTGGGGTCAGAGGCGGCGCTGCTTGGCTGTGAGC-3'
C289A/C290A reverse	5'-GCTCACAGCCAAGCAGCGCCGCCTCTGACCCCAGCAAGC-3'
C331A forward	5'-GTCATCATCTCCTCCTGAAGCGTTATCCGACTCCATCTGT-3'
C331A reverse	5'-ACAGATGGAGTCGGATAACGCTTCAGGAGGAGATGATGAC-3'

p62 forward_1BglII	5'-CTCAGATCTCGATGGCGTCGCTCACCG-3'
p62 forward_114BglII	5'-CTCAGATCTCGGCTCAGGAGGCGCCC-3'
p62 reverse_122SalI	5'-ACCGTCGACTCTACACCATGTTGCGGGGC-3'
p62 reverse_200SalI	5'-ACCGTCGACTCTAACCCATTTCCCATCCTGGC-3'
p62 reverse_440	5'-TCACAACGGCGGGGG-3'

Supplementary Table 1: Primers for mutagenesis and cloning used in this study.

**primer**	**Sequence**
p62 forward	5'-GCTGCCCTATACCCACATCT-3'
p62 reverse	5'-CGCCTTCATCCGAGAAAC-3'
Pkg1 forward	5'-TACCTGCTGGCTGGATGG-3'
Pkg1 reverse	5'-CACAGCCTCGGCATATTTCT-3'
HO-1 forward	5'-GGTCAGGTGTCCAGAGAAGG-3'
HO-1 reverse	5'-CTTCCAGGGCCGTGTAGATA-3'
Actin forward	5'-TAAGGCCAACCGTGAAAAG-3'
Actin reverse	5'-ACCAGAGGCATACAGGGACA-3'

Supplementary Table 2: Primers for qPCR used in this study.

Organism	Gene name	Protein ID
Homo sapiens (Human)	SQSTM1	Q13501|SQSTM_HUMAN
Pongo abelii (Sumatran orang-utan)	SQSTM1	Q5RBA5|SQSTM_PONAB
Macaca mulatta (Macaque)	SQSTM1	H9G1C8|H9G1C8_MACMU
Bos taurus (Cow)	SQSTM1	Q32PJ9|Q32PJ9_BOVIN
Mus musculus (Mouse)	Sqstm1	Q64337|SQSTM_MOUSE
Canis familiaris (Dog)	SQSTM1	F1P6L5|F1P6L5_CANFA
Cavia porcellus (Guinea pig)	SQSTM1	H0W6B1|H0W6B1_CAVPO
Gallus gallus (Chicken)	SQSTM1	F1NA86|F1NA86_CHICK
Sarcophilus harrisii (Tasmanian devil)	SQSTM1	G3W939|G3W939_SARHA
Anolis carolinensis (Carolina anole)	SQSTM1	G1KYX2|G1KYX2_ANOCA
Xenopus laevis (African clawed frog)	sqstm1	Q6PGS1|Q6PGS1_XENLA
Latimeria chalumnae (West indian Ocean coelacanth)	SQSTM1	H3AW10|H3AW10_LATCH
Drosophila melanogaster (Common fruit fly)	ref(2)P	P14199|REF2P_DROME
Caenorhabditis elegans (roundworm)	sqst-1	Q22436|Q22436_CAEEL

Supplementary Table 3: The p62 sequences that were used for multiple alignment.

Supplementary References

1. Bjorkoy, G. *et al.* p62/SQSTM1 forms protein aggregates degraded by autophagy and has a protective effect on huntingtin-induced cell death. *The Journal of cell biology* **171**, 603-614 (2005).
